# The mysterious Spotted Green Pigeon and its relation to the Dodo and its kindred

**DOI:** 10.1186/1471-2148-14-136

**Published:** 2014-07-16

**Authors:** Tim H Heupink, Hein van Grouw, David M Lambert

**Affiliations:** 1 Environmental Futures Research Institute, Griffith University, Nathan, Australia; 2 Natural History Museum, Tring, UK

**Keywords:** Spotted Green Pigeon, *Caloenas maculata*, Dodo, *Raphus cucullatus*, Extinct, Museum specimen, DNA extraction, Ancient DNA, Mini-barcode, Phylogeny

## Abstract

**Background:**

The closely related and extinct Dodo (*Raphus cucullatus*) and Rodrigues Solitaire (*Pezophaps solitaria*), both in the subfamily Raphinae, are members of a clade of morphologically very diverse pigeons. Genetic analyses have revealed that the Nicobar Pigeon (*Caloenas nicobarica*) is the closest living relative of these birds, thereby highlighting their ancestors’ remarkable migration and morphological evolution. The Spotted Green Pigeon (*Caloenas maculata*) was described in 1783 and showed some similarities to the Nicobar Pigeon. Soon however the taxon fell into obscurity, as it was regarded as simply an abnormal form of the Nicobar Pigeon. The relationship between both taxa has occasionally been questioned, leading some ornithologists to suggest that the two may in fact be different taxa. Today only one of the original two specimens survives and nothing is known about the origin of the taxon. Due to its potential close relationship, the Spotted Green Pigeon may hold clues to the historical migration, isolation and morphological evolution of the Dodo and its kindred.

**Results:**

We use ancient DNA methodologies to investigate the phylogeny and authenticity of the Spotted Green Pigeon. A novel extraction method with the ability to retain and purify heavily fragmented DNA is used to investigate two feathers from the sole surviving specimen. Maximum Likelihood phylogenetic analyses reveal that the Spotted Green Pigeon is a unique lineage and together with the Nicobar Pigeon, is basal to the Dodo and Rodrigues Solitaire.

**Conclusions:**

The distance observed for the Spotted Green Pigeon and Nicobar Pigeon is larger than that observed within other Pigeon species, indicating that the Spotted Green pigeon is a unique taxon, thereby also indicating it is a genuine addition to the list of extinct species. The phylogenetic placement of the Spotted Green Pigeon indicates that the ancestors of both *Caloenas* and therefore Raphinae displayed and shared the following traits: ability of flight, semi-terrestrial habits and an affinity towards islands. This set of traits supports the stepping stone hypothesis, which states that the Raphinae got to their respective localities by island hopping from India or Southeast Asia.

## Background

The Dodo (*Raphus cucullatus*) is an icon of extinction as well as extreme morphological evolution. The morphological distinctiveness of the Dodo has severely complicated the investigation of its relationship to other bird species. More recent genetic analyses have revealed that the Dodo from Mauritius and the closely related Rodrigues Solitaire (*Pezophaps solitaria*), both extinct flightless island endemics in the subfamily Raphinae, fall in a clade of morphologically very diverse Pigeon species. This extended Dodo clade includes, in order of closeness to the Dodo and the Rodrigues Solitaire, the genera *Caloenas*, *Goura* and *Didunculus *[1], of which all living species posses the ability to fly but show at least some degree of terrestrial habits. The genus *Caloenas* is represented by a single living species, the Nicobar Pigeon (*Caloenas nicobarica*), which has a circum-Indonesian distribution (from the Andaman to the Solomon Islands) and has a tendency to live on small and remote islands. The genus *Goura* is represented by three living species of Crowned Pigeon that are endemic to the island of New Guinea. Finally, the genus *Didunculus* is represented by a single living species, the Tooth-billed Pigeon *(D. strigirostris),* an endemic to the islands of Samoa.

**Figure 1:**
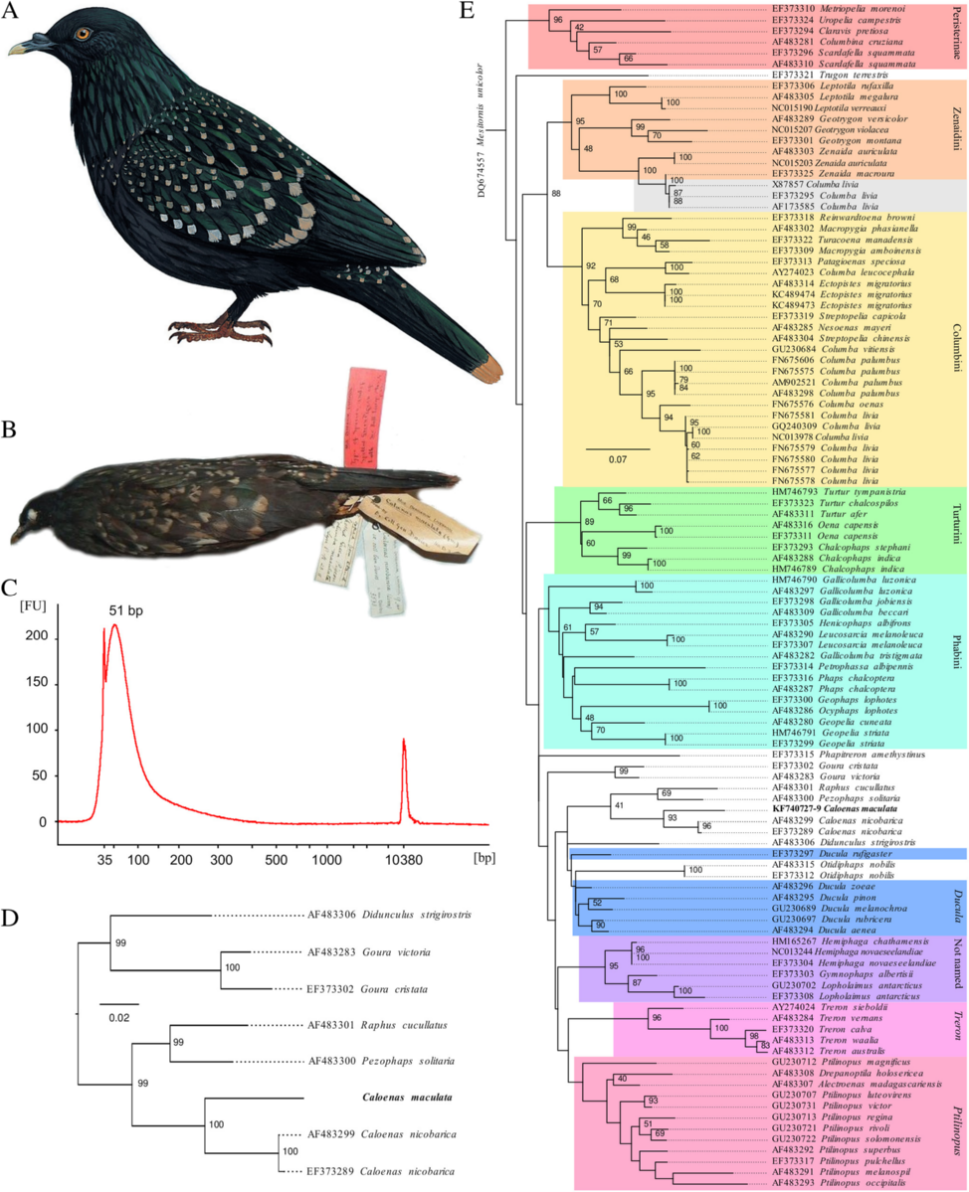
**The Spotted Green Pigeon, extracted DNA characteristics and phylogeny. (A)** Reconstruction of the Spotted Green or Liverpool Pigeon (courtesy of del Hoyo, J., Elliott, A., & Sargatal, J. eds. 2002. Handbook of the Birds of the World. Vol. 7. Jacamars to Woodpeckers. Lynx Edicions, Barcelona), **(B)** a picture of the sole surviving specimen (courtesy of Clemency Fisher and the World Museum, National Museums Liverpool), **(C)** Bioanalyzer plot for the first DNA extract highlighting the short fragmentary nature of the DNA (median 51 bp). 35 bp and 10380 bp peaks are markers. FU: fluorescent units., **(D)** Maximum likelihood tree for the concatenated Spotted Green Pigeon sequences and 12S sequences from members of the extended Dodo clade (as identified by Shapiro et al. [1]) and **(E)** Maximum likelihood tree for 106 Pigeon mitochondrial 12S sequences. The Spotted Green Pigeon (bold) clusters first with the Nicobar Pigeon and second with the Dodo and Rodrigues Solitaire. Previously identified Pigeon clades in the phylogeny are coloured. The reason for the clustering of three rock Pigeon sequences (*Columba livia*, grey box) with mourning doves (*Zenaida macroura*) is unclear, although hybridisation has been observed for these two species [5]. Bootstrap support values above 40 are indicated in the larger tree to allow for observation of the bootstrap value for the split between *Caloenas* and Raphinae, the dotted lines in both trees are there to associate the taxa with the appropriate tree tips.

The mysterious Spotted Green Pigeon (also known as the Liverpool Pigeon), *Caloenas maculata*, (Figure 1A) has fallen in to obscurity over time due to its unresolved species status, this species may however be closely related to and help understand the evolution of the Dodo. The Spotted Green Pigeon was first described in 1783 [2], only two known specimens existed at this time and were kept in the collections of Sir Joseph Banks and General Davies. Only the latter specimen remains and is currently kept in the World Museum (formerly Liverpool Museum), National Museums Liverpool (Figure 1B), hence it is also referred to as the Liverpool Pigeon. No locality data exist for either of the Spotted Green Pigeon specimens, both collectors however heavily focussed on the Oceania region, leading some authors to conclude the species most likely originated in this area. It has been suggested the pigeon resembles an extinct bird from Tahiti [3], but this has been disputed [4]. The specimen shows characteristics that have been associated with both arboreal and semi-terrestrial or island habits, e.g. fairly long tail feathers and short rounded wings respectively [3].

The green glossy feathers and elongated neck feathers (hackles) of the Spotted Green Pigeon have often been interpreted as evidence of a relationship with the Nicobar Pigeon. However, the extent and validity of this suggested relationship has often been questioned. Wagler [6] for example described the Spotted Green Pigeon as a juvenile specimen of the Nicobar Pigeon, whereas Rothschild & Hartert [7] considered the specimen an abnormality of this species. In both cases the authors regarded the two taxa as conspecific. Forbes [8] did recognise a grouping with *Caloenas*, but regarded both taxa as different species, i.e. heterospecific. Gibbs et al. [3] proposed a relatively distant relationship to the primarily Oceanic pigeon genera the Fruit Pigeons (*Ptilinopus*), the Imperial Pigeons (*Ducula*) and possibly the Mountain Pigeons (*Gymnophaps*), as opposed to *Caloenas*. The species status of the Spotted Green Pigeon thus remains unresolved and castes doubt on its listing as extinct in the BirdLife International IUCN Red List for birds [9].

To investigate the validity of this taxon and its relationship to the morphologically diverse extended Dodo clade we recovered and sequenced the DNA from two feathers from the sole surviving specimen of the Spotted Green Pigeon.

## Methods

All pre-PCR handling of the samples and their extracts was performed in a dedicated ancient DNA laboratory at Griffith University. Appropriate contamination controls and stringent sequencing protocols were in place to assure the authenticity of recovered sequences. Both feathers were extracted separately with associated extraction blanks. In both cases the feathers were finely cut up and extracted overnight at 56°C in 400 μl extraction buffer (0.45 M EDTA, 0.5% N-lauryl sarcosine, 1 mg/ml proteinase K). The resulting solution was centrifuged at maximum speed (20,000 × *g*) for 2 minutes. The supernatant was washed repeatedly with 1 ml equilibrated phenol (pH 7.5) until no more colouring was removed, each time vortexing for 5 seconds and separating both phases by centrifuging for 1 minute at maximum speed (20,000 × *g*). The aqueous phase was washed once with chloroform in the same fashion. The resulting solution was mixed with 10x buffer PB (Qiagen) for binding to Dneasy Blood & Tissue columns (Qiagen). We followed the kit’s instructions with the following modifications, note that buffer PB is supplied separately. The buffer PB extract solution was centrifuged in aliquots of 700 μl through one column per extract. The bound DNA was washed repeatedly with 700 μl buffer AW1 until no more colouring was removed from the filter. DNA was eluted in 50 μl of water. The DNA concentrations where measured for 1 μl of each extract and extraction blank using the Qubit 2.0 Fluorometer (Life Technologies) and the Bioanalyzer (Agilent), using the respective high sensitivity kits.

The designed 12S mini-barcodes and their associated primer pairs were investigated using a dataset of 240 bird species for which complete reference mitochondrial genomes were available (Addition file 1: Table S1). The three 12S mini-barcodes (64.3 bp average without primers) could in theory be amplified for most if not all birds, each consisting of a highly variable region on the 12 s ribosomal RNA region of the mitochondrial genome (69.8% average pairwise identity) flanked by highly conserved primer sites for amplification (98.2% average pairwise identity). All three 12S mini-barcodes were unique for the vast majority of species (221.7 on average).

The 25 μl 12S mini-barcode PCRs each contained 3 μl template, 1× High Fidelity PCR buffer, 2 mM MgCl2, 0.2 μM of each primer (Additional file 1: Table S1), 0.2 mM of each dNTP and 2.5 units of Platinum *Taq* High Fidelity (Invitrogen), thermocycling was as follows: 30 sec 94°C, 60× [15 sec 94°C, 30 sec annealing temperature (Additional file 1: Table S1), 30 sec 68°C], 1 min 68°C. Each PCR included a negative control and extraction blank. The amplified DNA was either isolated from a gel (in case of unspecific by-products) using the QIAquick Gel Extraction Kit (Qiagen) or cleaned with ExoSAP-IT (USB) according to manufacturer’s instructions. The BigDye V3.1 (Applied Biosystems) kit was used according to manufacturer’s instructions to sequence the DNA fragments. Each 12S mini-barcode was amplified three times independently for each sample and sequenced in the forward and reverse directions.

The reference dataset consisted of all matching 12S Pigeon DNA sequences (n = 106) available from GenBank (accession numbers in Figure 1E) and DQ674557 the Brown Mesite (*Mesitornis unicolor),* which was forced as an outgroup when reconstructing the phylogeny [10]. The dataset was aligned using MAFFT 7.130b [11] using the Q-INS-i algorithm that considers RNA structure with standard settings. A subset of this dataset was used for Figure 1D (see figure for accession numbers), the midpoint was used for rooting. Maximum Likelihood phylogenies for both datasets were reconstructed using RAxML 8.0.5 [12] with the GTRCAT model and 1000 bootstrap iterations, otherwise the standard settings were used.

According to the “Australian code for the care and use of animals for scientific purposes” this research did not require ethical approval since no living animals were involved.

## Results and discussion

An initial PCR targeting 138 bp mitochondrial DNA (including primers) failed to amplify any detectable product and highlighted the need for an approach that could retrieve short yet informative ancient DNA sequences. The DNeasy Blood & Tissue Kit normally provides the possibility to recover “DNA fragments as small as 100 bp”. In order to also recover the shorter DNA fragments the lysis step from this kit was replaced with a standard bone digestion followed by an organic extraction, the DNA was then bound to the Dneasy column using 10× buffer PB (supplied separately, this buffer contains a high concentration of isopropanol). The larger concentration of isopropanol in the final binding solution mixture appeared to promote the binding of shorter DNA molecules, a similar finding to Dabney et al. [13]. The mentioned kit includes wash buffer AW 1 which removes PCR inhibitors, we found that repeated washes with this buffer improved the removal of these substances, we also found that repeated phenol washes had a similar effect and removed other PCR inhibitors.This novel extraction method retained the very short fragmented DNA molecules (>30 bp), which were extracted and purified from two feathers. Measurements for the 50 μl extracts confirmed the low DNA quantity at 1.86 ng/μl and <0.10 ng/ul respectively, heavy fragmentation to 51 bp median length was observed for the first feather (Figure 1C). To avoid complications with assembly of longer loci using overlapping amplicons we designed three short but very informative mini-barcodes located on the mitochondrial genome’s 12S gene.

The three 12S mini-barcodes were each amplified three times independently for each of the two Spotted Green Pigeon samples, the products of which were sequenced in both directions (i.e. 36 sequences in total). All resulting sequences were consistent; the 12 sequences for each 12S mini-barcode assembled and showed no differences to each other, supporting their authenticity and making it unlikely that any of the characterised polymorphisms originated due to DNA damage. In addition BLAST searches (megablast nr/nt) confirmed that all three assembled sequences originated from a unique Pigeon taxon not yet previously characterised.

Both Maximum Likelihood phylogenies for the concatenated Spotted Green Pigeon 12S mini-barcodes identified the Nicobar Pigeon as the closest relative (Figure 1D,E), followed by the extinct Dodo and the Rodrigues Solitaire. The correct clustering of previously identified Pigeon groups in the Figure 1E phylogeny supports these inferences. The three 12S mini-barcodes showed an average 90.5% and 84.3% pairwise identity when compared to the Nicobar Pigeon and the Dodo respectively. The distance observed between the Spotted Green Pigeon and the Nicobar Pigeon in the phylogeny of Figure 1E (0.11 substitutions per site) is significantly greater than that observed *within* other Pigeon species; the average pairwise difference for taxa sharing the same genus and species name is 0.01 ± 0.01 substitutions per site (one sample *t*(13) = -35.97, *p* = 1.05 × 10^-14^). The observed distance however appears to correspond with that of other heterospecific Pigeon taxa, the distance does not significantly differ from the average pairwise distance between species sharing the same genus but not species name (0.09 ± 0.04 substitutions per site, one sample *t*(14) = -1.48 , *p* = 0.08).

## Conclusions

The presented novel extraction method opens up the possibility to characterise the shorter DNA fragments that previous extraction methods failed to retain, thereby making more ancient specimens possible targets for ancient DNA analysis. The 12S mini-barcodes resolve pigeon species and genera well and were originally designed to amplify for any bird species, making them a useful molecular tool for identifying bird specimens or remains with severely degraded and poorly preserved DNA. This research exemplifies how informative DNA mini-barcodes might, in combination with an efficient extraction methodology, be used to identify the evolutionary origin of museum specimens.

Most species in the extended Dodo clade show a characteristic mixture of terrestrial and arboreal traits and show a degree of affinity to islands, the same mixture of traits has been suggested for the Spotted Green Pigeon [3]. This observation, together with that of the suggested clustering with the Nicobar Pigeon, support the inferred phylogeny for the the Spotted Green Pigeon. Perhaps the morphological diversity in the extended Dodo clade combined with the observed relation to the Nicobar Pigeon explain the wealth of previously suggested relations for both *Caloenas* taxa. The presented statistics on the distances observed within and between species suggest that the Spotted Green Pigeon was relatively distantly related to the Nicobar Pigeon. The observed distances indicate that the Spotted Green Pigeon is a genuine taxon and therefore a good addition to the list of recently extinct species, making the last remaining specimen a genuine unicum.

The other species in the genus *Caloenas*, the extinct Kanaka Pigeon (*C. canacorum*) known from sub-fossil remains from New Caledonia and Tonga, shows no indications of a reduction in the ability to fly and its localities indicate an island hopping history [14,15]. This species is considered to be about 25% larger than the 40 cm Nicobar Pigeon and is thus unlikely to be conspecific with the 32 cm Spotted Green Pigeon. The Nicobar and Kanaka Pigeon may suggest a possible Oceanian or Southeast Asian origin for the Spotted Green Pigeon, the relation to the Raphinae however also opens up the possibility that the taxon originated from a location in the Indian Ocean. An island location seems most likely due to the habits of its relatives and fits its traits that have been associated with an island lifestyle.

The ancestor for the three *Caloenas* taxa most likely displayed the same characteristic traits as its descendants: the ability of flight, but semi-terrestrial habits and an affinity towards islands. The close relation of this particular ancestor with the ancestor of the Raphinae and the habits observed throughout the extended Dodo clade all point towards a similar set of traits for the ancestor of the Raphinae. This set of traits; ability of flight, semi-terrestrial habits and an affinity towards islands, all agree well with the stepping stone hypothesis [1]. This hypothesis suggests that the ancestors of the Dodo and the Rodrigues Solitaire travelled from island to island, starting somewhere in Southeast Asia or India and finishing in Mauritius and Rodrigues [16,17], the mentioned traits suggest that flight rather than any other form of transport was a primary contributor to this dispersal. As a result the unexpected addition of another member to the morphologically diverse extended Dodo clade supports the idea of the stepping stone hypothesis and contributes to our understanding of how the Dodo came to be such a remarkable example of distant isolation and subsequent morphological evolution.

## Availability of data

The DNA sequences characterised for this study are available on GenBank, accession numbers: KF740727-KF740729.

## Abbreviations

Bp: Base pairs; PCR: Polymerase chain reaction; EDTA: Ethylenediaminetetraacetic acid; IUCN: International Union for Conservation of Nature.

## Competing interests

The authors declare that they have no competing interests.

## Authors’ contributions

All authors contributed to the conception, design and coordination of the study. THH carried out the laboratory work, analysed the data, interpreted the results and drafted the manuscript. All authors read, edited and approved the final manuscript.

## Supplementary Material

Additional file 1: Table S1 Information about the 12S mini-barcodes, including primers, lengths and pairwise identities. Click here for file
